# Prognostic factors in hospitalized patients with COVID-19 pneumonia and effectiveness of prophylactic anticoagulant therapy: a single-center retrospective study

**DOI:** 10.1186/s12879-025-10666-3

**Published:** 2025-03-03

**Authors:** Xing He, Chun Zhang, Jiaqi Ji, Yang Liu, Wanjie Feng, Linjie Luo, Hong Fan, Lu Guo

**Affiliations:** 1 Department of Pulmonary and Critical Care Medicine, West China Hospital, Sichuan University, Chengdu, China; 2 State Key Laboratory of Respiratory Health and Multimorbidity, West China Hospital, Sichuan University, Chengdu, China; 3 Department of Pulmonary and Critical Care Medicine, School of Medicine, Sichuan Provincial People’s Hospital, University of Electronic Science and Technology of China, Chengdu, Sichuan China; 4 Department of Internal Medicine, Wenjiang District Third People’s Hospital of Chengdu, Chengdu, 611130 China; 5 Department of Critical Care Medicine, Wenjiang District People’s Hospital of Chengdu, Chengdu, Sichuan China

**Keywords:** Covid-19, Venous thromboembolism, Anticoagulant therapy, Hospitalization, Mortality

## Abstract

**Background:**

COVID-19 pneumonia patients encounter the potential risk of venous thromboembolism (VTE) and mortality during hospitalization. This study aimed to analyzed risk factors of all-cause mortality in hospitalized patients with COVID-19 pneumonia, and investigated the effectiveness of prophylactic anticoagulation and hospital stays on the mortality in hospitalized patients with nonVTE.

**Methods:**

We retrospectively analyzed all COVID-19 pneumonia patients who were admitted to our medical center from December 2022 to January 2023. Clinical data and outcome events were collected from patients’ electronic medical records. Cox regression was used to identify poor prognostic factors of COVID-19 pneumonia patients with VTE and nonVTE. Landmark analysis was conducted to identify time points of hospital stays between anticoagulation treatment and in-hospital survival outcomes in COVID-19 pneumonia patients with nonVTE. Binary logistic regression analysis was performed to investigate factors related to prolonged hospital stays.

**Results:**

Among 2,520 COVID-19 pneumonia patients, 1047 received prophylactic anticoagulation and 76 complicated with VTE during hospitalization. Survival curve analysis showed no statistically significant difference in mortality between COVID-19 pneumonia patients with VTE and nonVTE in prophylactic anticoagulant group (*P* = 0.63). Multivariate cox regression analysis revealed that male(HR = 1.398, 95%CI= [1.021,1.915]), BMI (HR = 0.935, 95%CI= [0.900,0.972]), lymphocytes (HR = 0.576, 95%CI= [0.409,0.809]), platelets (HR = 0.997, 95%CI= [0.995,0.999]), albumin (HR = 0.950, 95%CI= [0.926,0.975]), lactate dehydrogenase (HR = 1.001, 95%CI= [1.001,1.002]) were risk factors for mortality in COVID-19 pneumonia patients with nonVTE, while sCRP (HR = 1.010, 95%CI= [1.004,1.015]), anticoagulant therapy (HR = 0.247, 95%CI= [0.096,0.632]) were risk factors for mortality in COVID-19 pneumonia patients with VTE. Landmark analysis showed that for the hospital stays of 11 days, the difference in the impact of prophylactic anticoagulation on mortality was statistically significant in COVID-19 pneumonia patients with nonVTE (≤ 11days, *P* = 0.014; > 11days, *P* = 0.01). CVD (OR = 1.717, 95%CI= [1.248,2.363]), CRD (OR = 1.605, 95%CI= [1.133,2.274]), sCRP (OR = 1.003, 95%CI= [1.000,1.006]), Alb (OR = 0.959, 95%CI = [0.932,0.987]) and use of glucocorticoid (OR = 1.428, 95%CI= [1.057,1.930]) were independent factors associated with hospital stays > 11 days in anticoagulant group.

**Conclusions:**

This study indicated that Male, lower BMI, peripheral blood lymphocytes, platelets, albumin and elevated lactate dehydrogenase were associated with poor hospitalisation outcomes in COVID-19 pneumonia patients with nonVTE. As for COVID-19 pneumonia patients with VTE, poor hospitalisation outcomes were associated with elevated sCRP levels and no given anticoagulant therapy. No significant difference in mortality between hospitalized COVID-19 pneumonia patients with VTE and nonVTE when receiving prophylactic anticoagulation. Prolonged hospital stays (> 11 days) may limit the effectiveness of prophylactic anticoagulation on lower in-hospital mortality for COVID-19 pneumonia patients with nonVTE.

**Supplementary Information:**

The online version contains supplementary material available at 10.1186/s12879-025-10666-3.

## Introduction

The coronavirus disease 2019 (COVID-19) is an infectious disease caused by severe acute respiratory syndrome coronavirus 2 (SARS-CoV-2). Patients with COVID-19 experience varying degrees of clinical symptoms, inflammatory responses and organ dysfunction. Approximately 10% of patients develop hypoxic COVID-19 pneumonia, with 3% progressing to severe cases [1]. Acute respiratory distress syndrome occurs in 15–30% of hospitalized patients with COVID-19 pneumonia, of which the overall mortality rate is 26% [2, 3]. Given that COVID-19 pneumonia has been routinely managed, timely identification of high-risk patients during hospitalization can facilitate early interventions aimed at improving prognosis.

The inflammatory response triggered by COVID-19 significantly contributes to coagulation dysfunction [4], further increasing the risk of thrombosis. A cohort study in French showed that the prevalence of venous thromboembolism (VTE) in patients with COVID-19 was 5.2% [5]. The incidence of VTE varies considerably upon hospitalization, with an incidence rate of 2.2% in non-intensive care unit (ICU) patients and 27.0% in ICU patients [6]. Among hospitalized COVID-19 patients with VTE, the 10-day fatality rate is 9.1%, rising to 19% in ICU patients [7]. Anticoagulant therapy plays a key role in the prevention and treatment of VTE, yet patients undergoing anticoagulation still encounter the risk of thrombosis and mortality during hospitalization [8, 9]. Consequently, it is of great significance to evaluate the effectiveness of anticoagulant therapy on the prognosis of hospitalized patients. Studies on the relationship between anticoagulation and prognosis in hospitalized patients with COVID-19 has largely focused on populations infected with SARS-CoV-2, including COVID-19 pneumonia [10–12]. In fact, compared to patients with mild SARS-CoV-2 infection who do not present pneumonia, those with COVID-19 pneumonia show more severe inflammatory responses and an elevated risk of adverse effects, hospitalized patients with COVID-19 pneumonia exhibit a significantly higher mortality risk than individuals with community-acquired pneumonia [13], underscoring the necessity for more attention to this population. Besides, anticoagulants can effectively reduce the all-cause mortality of hospitalized patients with COVID-19 pneumonia [14], while increasing anticoagulant doses may heighten the risk of bleeding [15]. Certain patients are at risk for thrombosis irrespective of their chosen anticoagulant therapy, as COVID-19 pneumonia can contribute to this condition [16]. In any case, anticoagulant therapy is recommended for COVID-19 pneumonia patients complicated with VTE, whereas there is still a lack of research evidence on the effect of drug type, treatment duration in anticoagulant regimen on inpatient survival outcomes for COVID-19 pneumonia patients with nonVTE [17]. Meanwhile, the relationship between length of hospital stays and mortality in patients with Covid-19 pneumonia that receiving prophylactic anticoagulant therapy remains unclear.

Therefore, our study analyzed risk factors of all-cause mortality in hospitalized patients with COVID-19 pneumonia, comparing those with VTE to those with nonVTE. Additionally, we investigated the effectiveness of prophylactic anticoagulation and hospital stays on the mortality in hospitalized patients with nonVTE.

## Materials and methods

### Study design and object

This is a single-center retrospective study conducted at Sichuan Provincial People’s Hospital, where patients were hospitalized due to COVID-19 from December 2022 to January 2023.This study was approved by the Ethics Committee of Sichuan Provincial People’s Hospital (Process No. 2024 − 570). The Ethics Committee of Sichuan Provincial People’s Hospital agreed to exempt patients from written informed consent. In accordance with the World Health Organization standards [18], all included COVID-19 patients had positive results for SARS-CoV-2 nucleic acid, as determined by reverse transcription-polymerase chain reaction from throat or nasopharyngeal swabs upon admission. These tests were repeated within 24 h to exclude false-positive results. The diagnosis of COVID-19 pneumonia was based on chest CT scans, revealing localized or multi-lobar ground-glass opacities, consolidations, and reticular opacities. Preliminary imaging evaluations were performed by radiologists, who collaborated with clinicians to confirm the diagnosis of COVID-19 pneumonia. All patients suspected of VTE during hospitalization underwent CT pulmonary angiography (CTPA) and color ultrasonography of the upper and lower extremities to assess the presence of VTE events.

### Eligibility criteria

Exclusion criteria were as follows: (1) presence of malignant tumors; (2) age < 18 years; (3) confirmed diagnosis of any VTE events upon admission. (4) Since this study specifically targeted patients with COVID-19 pneumonia, all COVID-19 patients without evidence of pneumonia on chest CT were excluded.

### Data collection

All data were extracted from patients’ electronic medical records, which were collected independently by four physicians.

General clinical data encompassed age, gender, body mass index (BMI), smoking history, surgical history within 3 months, cardiovascular disease (CVD), type 2 diabetes mellitus (DM), chronic lung disease (CLD), chronic renal disease (CRD), autoimmune disease (AID), and hospital stays.

Laboratory data contained peripheral blood-related results, covering white blood cell (WBC), neutrophil (Neu), lymphocytes (Lym), neutrophil/lymphocyte ratio (NLR), platelets (Plt), hypersensitive C-reactive protein (sCRP), high-sensitivity cardiac troponin T (Hs-cTnT), urea, creatinine (Cr), albumin (Alb), lactate dehydrogenase (LDH), creatine kinase (CK), brain natriuretic peptide (BNP), fibrinogen (Fib), D-dimer (D2), interleukin-6 (IL-6), etc. Laboratory data were acquired from the initial examination results at the time of admission.

Therapeutic regimen data included anti-COVID-19 drugs (Azvudine and Nirmatrelvir/Ritonavir), anticoagulants and duration of anticoagulant therapy (AT).

Our study meticulously documented the number of missing data for each parameter.

### Treatment strategy

The Padua score was used to assess the risk of VTE in all COVID-19 pneumonia patients admitted to general wards [19]. For COVID-19 pneumonia patients with a Padua score ≥ 4 and no evidence of thrombosis, prophylactic anticoagulation was administered after excluding contraindications, tailored to the patient’s condition and bleeding risk (excluding patients already on anticoagulant therapy). For nonVTE patients admitted to ICU, prophylactic anticoagulation treatment was administered directly [20]. Anticoagulants included low-molecular-weight heparin (enoxaparin and nadroparin), warfarin, novel oral anticoagulants (rivaroxaban, dabigatran etexilate, apixaban), or low-molecular-weight heparin combined with novel oral anticoagulants.

All patients underwent their assessment of initial Padua score and bleeding risk within 24 h of admission. Re-evaluations for any changes in patients were conducted during hospitalization, with corresponding adjustments made to the anticoagulation strategy.

Upon admission, all patients received oxygen therapy, symptomatic treatment, comorbidity therapy, maintenance of organ function, nutritional support and nursing care. Due to the significant medical burden resulting from the abrupt increase in COVID-19 pneumonia cases over a short period, anti-COVID-19 drugs were insufficient to meet the demand of all patients in the short term. If evidence of an additional etiological infection was identified during hospitalization, the patient would receive appropriate anti-infective therapy.

### Outcomes

The outcomes focused on the incidence of VTE and all-cause mortality during hospitalization. Secondary outcome: ICU admission and bleeding events.

### Sample size

To ensure the stability of the regression model and the reliability of results, the most stringent empirical rule was employed, that is, the sample size was set to more than 10 times the number of variables [21]. The calculated outcome indicated that the sample size in each group conformed to the requirement of statistical analysis.

### Statistical methods

Continuous variables were assessed for normality using Shapiro-Wilk test. Normally distributed data were expressed as mean ± standard deviation, with group differences analyzed by t-test; non-normally distributed data were reported as median (interquartile range), with group differences evaluated by Mann-Whitney U. Chi-square test was employed to analyze differences in binary categorical variables. Kaplan-Meier survival curve was utilized to evaluate the mortality associated with various grouping factors, and the difference in mortality between groups was assessed with the Log-rank method. If the survival curves did not intersect, it indicated that the categorical covariates of the group satisfied the proportional hazards assumption. The Schoenfeld residual method was applied to detect continuous variables. If there was no correlation trend between the residual and time, it was considered that the continuous variable was accord with the assumption of proportional hazards assumption. Additionally, all continuous variables underwent multicollinearity examinations. Variables that met the proportional hazards assumption and showed no collinearity were included in Cox proportional hazards regression model to explore factors associated with survival outcomes of COVID-19 pneumonia patients with VTE and nonVTE. Landmark analysis was conducted to identify time points influencing the association between anticoagulation treatment and in-hospital survival outcomes in COVID-19 pneumonia patients with nonVTE. Binary logistic regression analysis was performed to investigate factors related to prolonged hospital stays. The type of the missing data was completely random, and the sample size satisfied the requirements for difference analysis and regression model; following the 80% rule, we did not perform any processing on parameters with less than 20% missing data [22]. However, since creatine kinase, brain natriuretic peptide and interleukin-6 exhibited more than 20% missing data, we excluded these three parameters from our statistical analyses to maintain the stability of the regression model and ensure the reliability of our results. SPSS 26.0 software was applied for inter-group difference, collinearity test of continuous variables and regression analysis. Graphpad Prism 10 was employed for flow charts and forest plots. Survival curves and Log-rank tests were generated using the ggsurvplot package in RStudio. Landmark analysis and statistical analysis via Schoenfeld residual method were performed using the jskm and ggcoxzph packages, respectively. *P* < 0.05 was considered statistically significant.

**Fig. 1:**
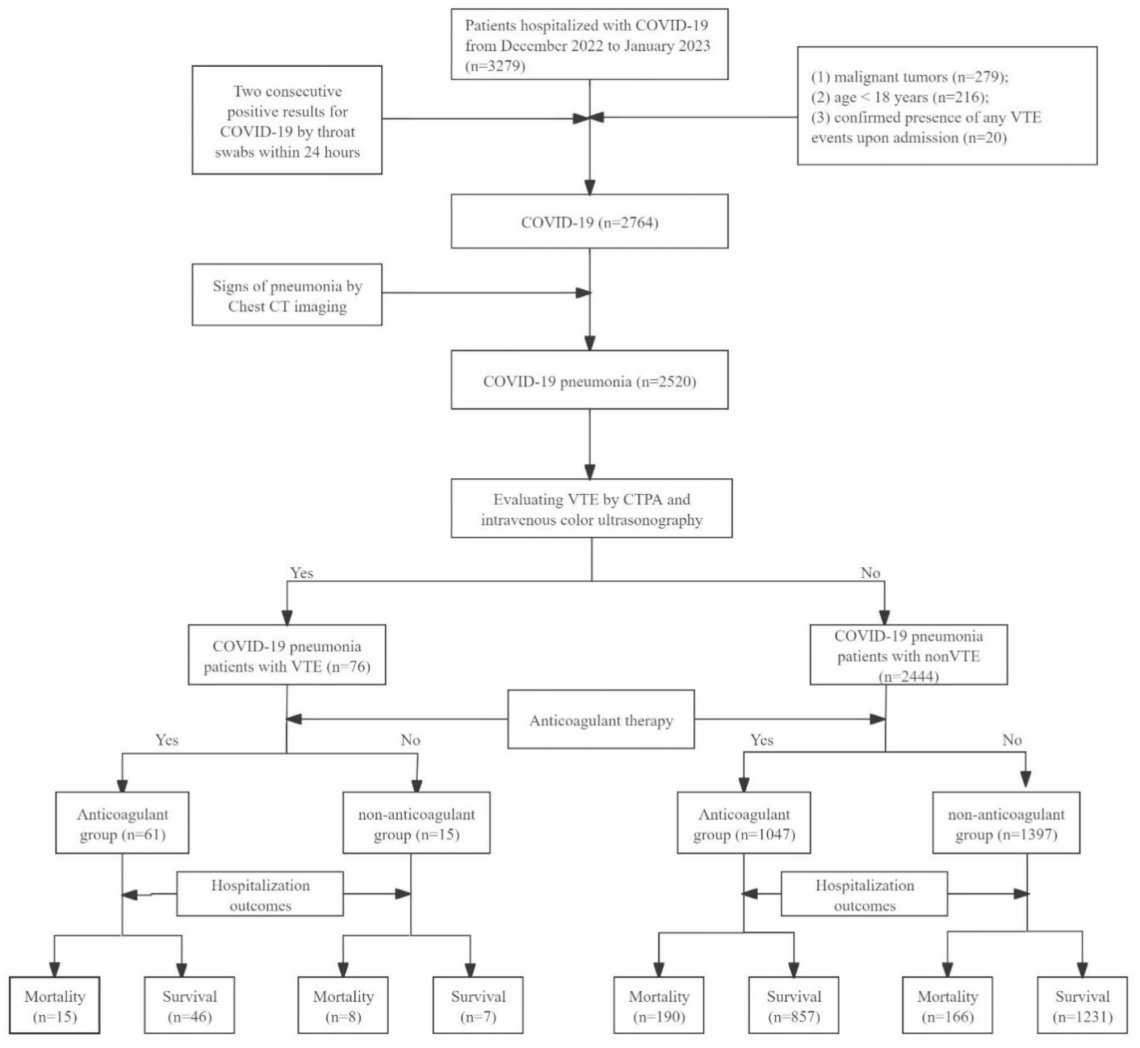
Flow chart of this study

## Results

### Screening process of included patients

This study screened a total of 3,279 patients hospitalized with COVID-19 from December 2022 to January 2023. Initially, we excluded 279 patients with malignant tumors, 216 patients under 18 years of age and 20 patients with any confirmed VTE at admission. Subsequently, the chest CT imaging results of the remaining 2,764 COVID-19 patients were evaluated, leading to the identification of 2,520 patients who met the criteria for COVID-19 pneumonia. Through a review of electronic medical records and imaging findings (CTPA and intravenous color ultrasonography), 76 COVID-19 pneumonia patients were diagnosed with VTE, and 2,444 were categorized as nonVTE. Among those with VTE, therapeutic anticoagulation was administered to 61 patients resulting in 15 deaths (24.6%); conversely, among remaining 15 patients who did not use any anticoagulants— due to various bleeding events (*n* = 12) or unknown reasons (*n* = 3)— there were 8 deaths (53.3%). Among COVID-19 pneumonia patients with nonVTE, 1047 received anticoagulant therapy, with 190 deaths (18.1%); 1397 patients did not receive any anticoagulant, with 166 deaths (11.8%) (Fig. 1).

### Comparison of clinical data and mortality between COVID-19 pneumonia patients with VTE and nonvte

The results indicated that COVID-19 pneumonia patients in VTE group exhibited significantly higher levels of age, hospital stays, Neu, NLR, sCRP, Hs-cTnT, Urea, LDH, BNP and D2 compared to those in nonVTE group (*P* < 0.05) (Table 1). Conversely, levels of Lym, Plt and Alb were significantly lower in VTE group than in nonVTE group (*P* < 0.05). The proportions of patients with prior VTE history, ICU admission, use of anticoagulants, use of glucocorticoid and in-hospital mortality were significantly higher in VTE group compared to nonVTE group (*P* < 0.05) (Table 1).

No significant differences were observed between two groups regarding gender, BMI, smoking history, surgical history within 3 months, CVD, DM, CLD, CRD, AID, WBC, Cr, CK, Fib, IL-6, use of anti-COVID-19 drugs and the proportion of patients with bleeding events (*P* > 0.05) (Table 1).

**Fig. 2:**
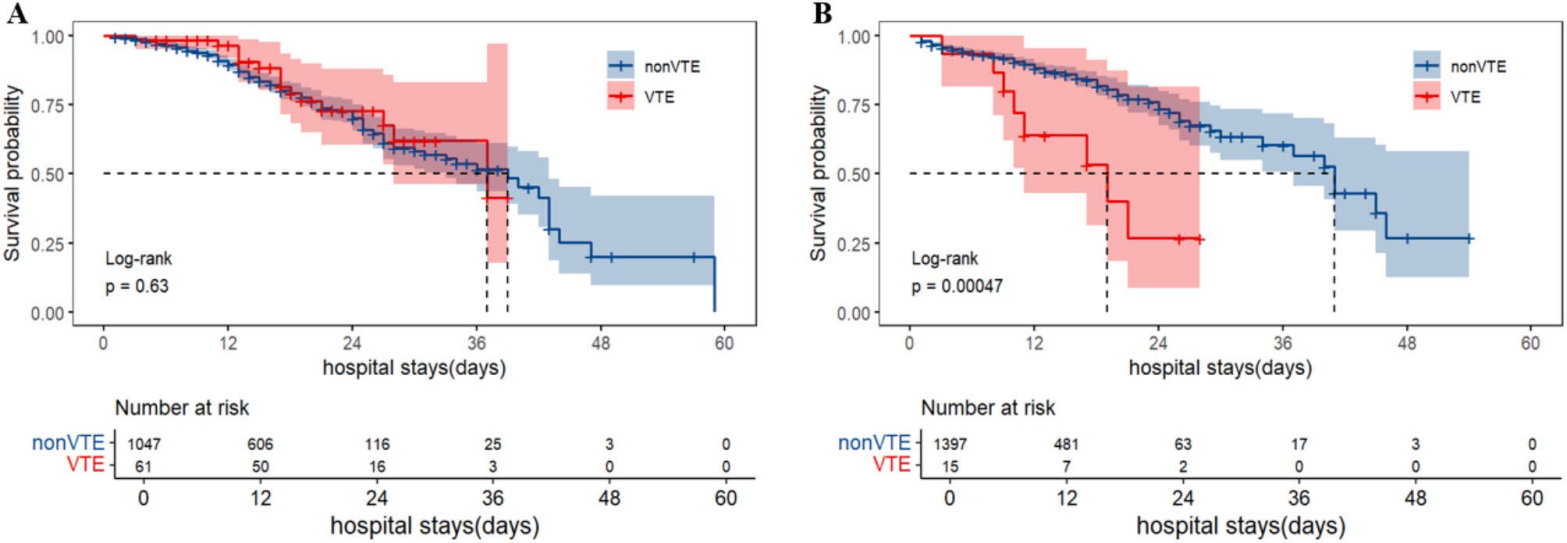
Comparison of mortality between COVID-19 pneumonia patients with VTE and nonVTE. **A**: Comparison of mortality between COVID-19 pneumonia patients with VTE and nonVTE in anticoagulant group; **B**: Comparison of mortality between COVID-19 pneumonia patients with VTE and nonVTE in non-anticoagulant group. COVID: coronavirus disease 2019; VTE: venous thromboembolism

Survival curve analysis showed no statistically significant difference in mortality between COVID-19 pneumonia patients with VTE and nonVTE in anticoagulant group (Log-rank test, *P* = 0.63) (Fig. 2A). In contrast, a significant difference was noted in mortality between COVID-19 pneumonia patients with VTE and nonVTE in non-anticoagulant group (Log-rank test, *P* < 0.001) (Fig. 2B).

**Table 1:** Comparison of clinical characteristics and laboratory data between COVID-19 pneumonia patients with VTE and nonvte

	COVID-19 pneumonia (*n* = 2520)	COVID-19 pneumonia with VTE (*n* = 76)	Missing data	COVID-19 pneumonia with nonVTE (*n* = 2444)	Missing data	Z/χ^2^	*P*
**General data**							
Age	73[59–83]	80.5[71.3–86.0]	NA	72[59–82]	NA	-4.841	< 0.001
Male	1570(62.3)	53(69.7)	NA	1517(62.1)	NA	1.845	0.174
BMI	23.42[20.90-25.92]	23.72[21.35–26.14]	17	23.39[20.89–25.91]	357	-0.599	0.549
Smoking history	584(23.2)	23(30.3)	NA	561(23)	NA	2.212	0.137
Prior VTE history	31(1.2)	4(5.3)	NA	27(1.1)	NA	7.347	0.007
Surgical history within 3 months	25(1)	1(1.3)	NA	24(1)	NA	< 0.001	1.000
CVD	1485(58.9)	52(68.4)	NA	1433(58.6)	NA	2.917	0.088
DM	701(27.8)	20(26.3)	NA	681(27.9)	NA	0.088	0.767
CLD	532(21.1)	16(21.1)	NA	516(21.1)	NA	< 0.001	0.990
CRD	531(21.1)	20(26.3)	NA	511(20.9)	NA	1.296	0.255
AID	135(5.4)	4(5.3)	NA	131(5.4)	NA	0.001	0.971
ICU admission	373(14.8)	22(28.9)	NA	351(14.4)	NA	12.420	< 0.001
Hospital stays (Day)	11[7–16]	17[11–23]	NA	11[7–15]	NA	-6.775	< 0.001
**Laboratory data**							
WBC	6.31[4.68–8.56]	6.76[5.28–8.96]	NA	6.29[4.65–8.54]	3	-1.694	0.090
Neu	4.67[3.18–6.94]	5.36[3.76–8.22]	NA	4.65[3.14–6.89]	3	-2.639	0.008
Lym	0.84[0.54–1.26]	0.67[0.41–0.91]	NA	0.85[0.54–1.27]	3	-3.871	< 0.001
NLR	5.41[2.97–10.80]	8.00[4.83–17.46]	NA	5.34[2.93–10.62]	3	-4.109	< 0.001
Plt	174[125–238]	145[109–200]	NA	175[125–239]	4	-2.772	0.006
sCRP	29.9[7.3–73.7]	54.0[23.6-114.3]	NA	28.6[7.1–72.5]	137	-4.193	< 0.001
Hs-cTnT	18.0[9.0-36.9]	23.3[17.7–60.7]	2	17.5[8.7–36.4]	449	-4.131	< 0.001
Urea	6.50[4.70–9.95]	7.51[5.52–12.57]	NA	6.42[4.68–9.89]	18	-2.655	0.008
Cr	82.9[64.5–115.0]	82.1[65.0-129.0]	NA	82.9[64.4–115.0]	18	-0.385	0.701
Alb	35.9[32.0-39.4]	32.5[29.2–37.3]	NA	36.0[32.2–39.4]	16	-4.558	< 0.001
LDH	264[209–344]	342[261–417]	NA	262[208–340]	106	-4.818	< 0.001
CK	75[46–146]	86[50–163]	12	74[45 − 15]	701	-1.059	0.289
BNP	59.5[21.7-150.6]	111.0[44.9-283.5]	NA	56.9[20.8-144.3]	516	-4.492	< 0.001
Fib	4.58[3.56–5.73]	4.32[3.59–5.29]	NA	4.58[3.56–5.76]	62	-1.199	0.230
D2	0.84[0.38-2.00]	1.91[0.95–5.14]	NA	0.82[0.37–1.94]	182	-6.349	< 0.001
IL-6	20.97[6.13–70.81]	37.04[8.49–89.17]	29	20.39[5.96–68.71]	1549	-1.658	0.097
**Treatment strategy**							
Untreated	1412(56.0)	15(19.7)	NA	1397(57.2)	NA	44.444	< 0.001
Low-molecular-weight heparin	1084(43.0)	61(80.3)		1023(41.9)			
Warfarin	16(0.7)	0(0)		16(0.7)			
Novel oral anticoagulant	8(0.3)	0(0)		8(0.3)			
glucocorticoid	1028(40.8)	53(69.7)	NA	975(39.9)	NA	27.180	< 0.001
**Anti-COVID-19 drug**							
Untreated	1401(55.6)	32(42.1)	NA	1369(56.0)	NA	5.957	0.114
Azvudine	865(34.3)	33(43.4)	NA	832(34.0)	NA		
Nirmatrelvir/Ritonavir	186(7.4)	8(10.5)	NA	178(7.3)	NA		
Azvudine + Nirmatrelvir/Ritonavir	68(2.7)	3(3.9)	NA	65(2.7)	NA		
**Outcomes**							
VTE	NA	76	NA	NA	NA	NA	NA
Pulmonary embolism		1(1.3)					
Deep vein thrombosis		6(7.9)					
Pulmonary embolism + Deep vein thrombosis		69(90.8)					
Bleeding event	70(2.8)	5(6.6)	NA	65(2.7)	NA	2.867	0.090
In-hospital mortality	379(15.0)	23(30.3)	NA	356(14.6)	NA	14.213	< 0.001

COVID: coronavirus disease 2019; BMI: body mass index; CVD: cardiovascular disease; DM: diabetes mellitus; CLD: chronic lung disease; CRD: chronic renal disease; AID: autoimmune disease; ICU: intensive care unit; WBC: white blood cell; Neu: neutrophil; Lym: lymphocytes; NLR: neutrophil/lymphocyte ratio; Plt: platelets; sCRP: hypersensitive C-reactive protein; Hs-cTnT: high-sensitivity cardiac troponin T; Cr: creatinine; Alb: albumin; LDH: lactate dehydrogenase; CK: creatine kinase; BNP: brain natriuretic peptide; Fib: fibrinogen; D2: D-dimer; IL-6: interleukin-6; VTE: venous thromboembolism; NA: not applicable

### Comparison of clinical data in COVID-19 pneumonia patients with nonvte between mortality and survival groups and exploration of risk factors for all-cause mortality

Among COVID-19 pneumonia patients with nonVTE, the mortality group exhibited significantly higher levels of age, WBC, Neu, NLR, sCRP, Hs-cTnT, urea, Cr, LDH, CK, BNP, Fib, D2 and IL-6 compared to the survival group (*P* < 0.05) (Table 2). Conversely, this group also demonstrated significantly lower BMI, hospital stays, Lym, Plt and Alb than the survival group (*P* < 0.05) (Table 2). In mortality group, the proportions of male patients, prior VTE history, CVD, DM, CLD, CRD, ICU admission, use of anticoagulants, glucocorticoid or anti-COVID-19 drugs, and bleeding events were significantly higher than in survival group (*P* < 0.05) (Table 2).

**Fig. 3:**
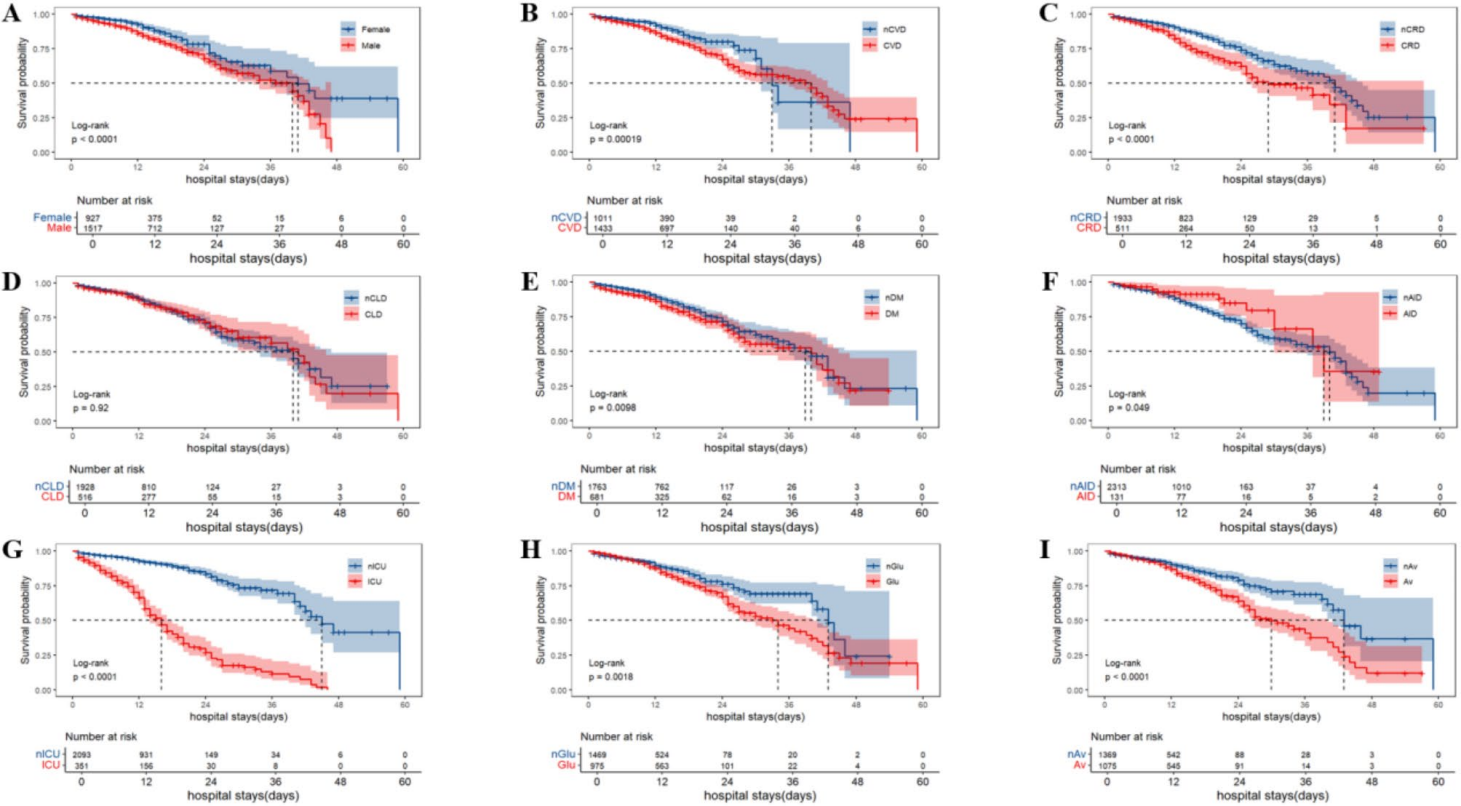
The effect of various factors on mortality in COVID-19 pneumonia patients with nonVTE by Kaplan-Meier curves and Log-rank test (Unadjusted) (**A**: Gender; **B**: CVD; **C**: CRD; **D**: CLD; **E**: DM; **F**: AID; **G**: ICU; **H**: Glu; **I**: Av). CVD: cardiovascular disease; CLD: chronic lung disease; CRD: chronic renal disease; DM: diabetes mellitus; AID: autoimmune disease; ICU: intensive care unit; Glu: glucocorticoid; Av: antiviral; VTE: venous thromboembolism; COVID: coronavirus disease 2019

Survival curve analysis showed significant differences in mortality rates among COVID-19 pneumonia patients with nonVTE in male gender, CRD, ICU admission, the use of glucocorticoid and the use of anti-COVID-19 drugs (Log-rank test, *P* < 0.05) (Fig. 3A-I). By Kaplan-Meier curve (Fig. 3A-I) and Schoenfeld residual method (Supplementary Fig. 1), all target variables satisfied the proportional hazards assumption. Collinearity diagnostics were performed on all continuous variables (Supplementary Table 1), and variables with a high collinearity risk were excluded.

**Fig. 4:**
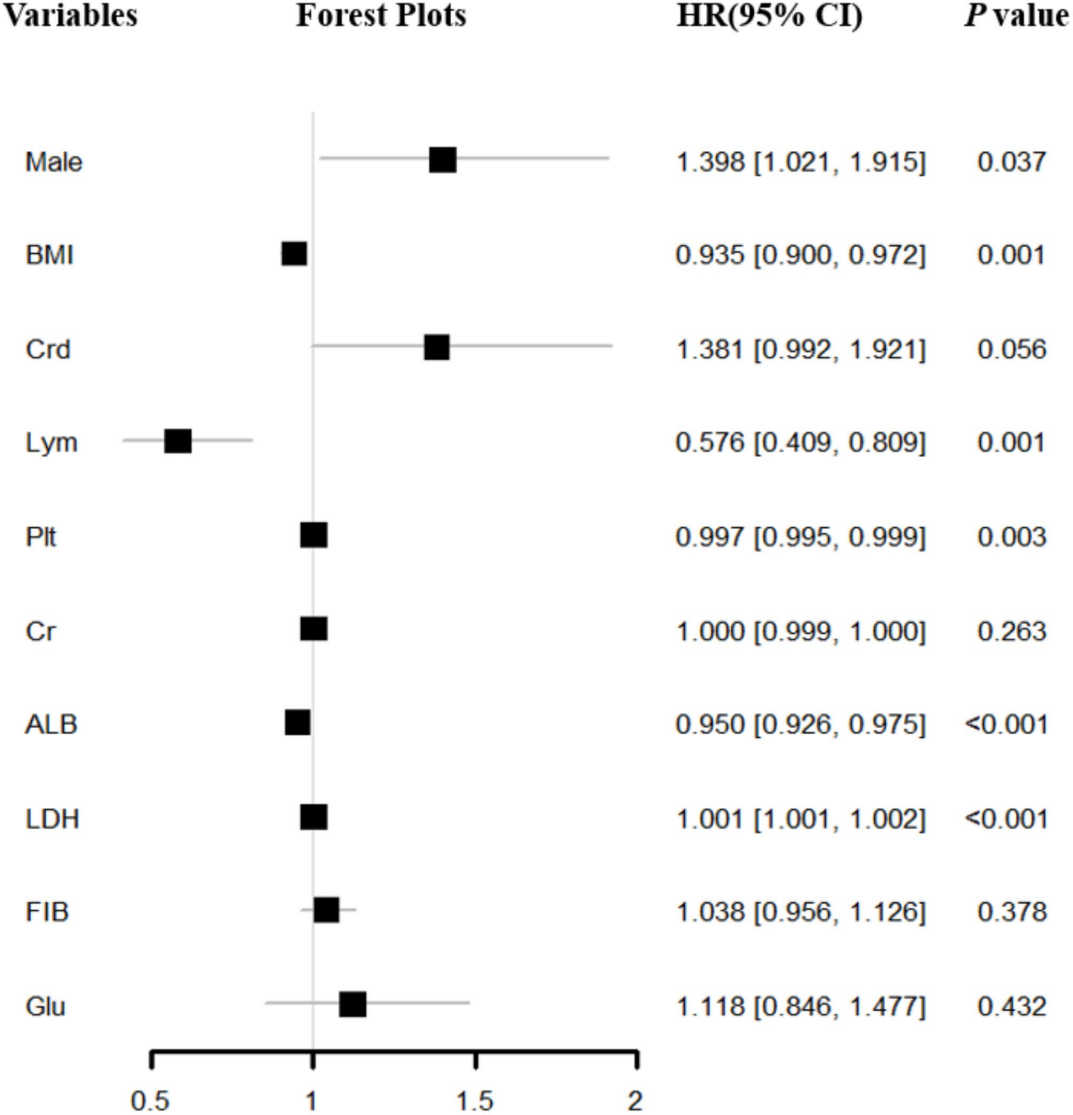
Forest plots of Cox proportional hazards regression model for all-cause mortality in hospitalized COVID-19 pneumonia patients with nonVTE BMI: body mass index; CRD: chronic renal disease; Lym: lymphocytes; Plt: platelets; Cr: creatinine; Alb: albumin; LDH: lactate dehydrogenase; Fib: fibrinogen; Glu: glucocorticoid; COVID: coronavirus disease 2019; VTE: venous thromboembolism

Eligible potential variables were incorporated into a multivariate Cox regression analysis, revealing that male (HR = 1.398, 95%CI= [1.021,1.915], *P* = 0.037), BMI (HR = 0.935, 95%CI= [0.900,0.972], *P* = 0.001), Lym (HR = 0.576, 95%CI= [0.409,0.809], *P* = 0.001), Plt (HR = 0.997, 95%CI= [0.995,0.999], *P* = 0.003), Alb (HR = 0.950, 95%CI= [0.926,0.975], *P* < 0.001), LDH (HR = 1.001, 95%CI= [1.001,1.002], *P* < 0.001) were risk factors for in-hospital all-cause mortality in COVID-19 pneumonia patients with nonVTE (Fig. 4).

**Table 2:** Clinical characteristics and laboratory results of poor prognosis in COVID-19 pneumonia patients with nonvte

	Mortality group (*n* = 356)	Missing data	Survival group (*n* = 2088)	Missing data	Z/χ^2^	*P*
**General data**						
Age	82[72–88]	NA	71[58–81]	NA	-12.361	< 0.001
Male	266(74.7)	NA	1251(59.9)	NA	28.317	< 0.001
BMI	22.44[19.60-24.92]	116	23.44[21.05–25.95]	241	-4.660	< 0.001
Smoking history	92 (25.8)	NA	469 (22.5)	NA	1.966	0.161
Prior VTE history	9(2.5)	NA	18(0.9)	NA	6.277	0.012
Surgical history within 3 months	3(0.8)	NA	21(1)	NA	< 0.001	1.000
CVD	267(75)	NA	1166(55.8)	NA	46.020	< 0.001
DM	132(37.1)	NA	549(26.3)	NA	17.602	< 0.001
CLD	93(26.1)	NA	423(20.3)	NA	6.281	0.012
CRD	122(34.3)	NA	389(18.6)	NA	44.985	< 0.001
AID	16(4.5)	NA	115 (5.5)	NA	0.616	0.433
ICU admission	181(50.8)	NA	170(8.1)	NA	450.9	< 0.001
Hospital stays (Day)	10.5[4.0–16.0]	NA	11.0[7.0–15.0]	NA	-2.385	0.017
**Laboratory data**						
WBC	8.20[5.77–12.18]	NA	6.13[4.57–8.14]	3	-9.280	< 0.001
Neu	7.09[4.47–10.86]	NA	4.43[3.04–6.37]	3	-11.453	< 0.001
Lym	0.57[0.34–0.88]	NA	0.91[0.59–1.34]	3	-12.112	< 0.001
NLR	12.27[5.97–23.47]	NA	4.85[2.76–8.88]	3	-15.053	< 0.001
Plt	149[101–209]	NA	178[130–243]	4	-6.510	< 0.001
sCRP	79.5[37.6-134.4]	19	22.4[5.9–60.4]	118	-14.157	< 0.001
Hs-cTnT	43.3[24.8–96.1]	24	14.7[7.8–28.3]	425	-17.344	< 0.001
Urea	10.42[7.28–17.8]	1	6.04[4.50–8.78]	17	-15.206	< 0.001
Cr	112.5[79.5-181.3]	1	80.0[62.9–106.0]	17	-10.608	< 0.001
Alb	32.5[29.4–36.2]	1	36.6[32.8–39.8]	15	-11.543	< 0.001
LDH	363[267–532]	21	252[203–320]	85	-14.053	< 0.001
CK	133[62–326]	117	70[43–129]	584	-8.197	< 0.001
BNP	155.4[62.4-412.5]	30	46.8[18.5-113.3]	486	-13.284	< 0.001
Fib	4.74[3.80–6.06]	8	4.55[3.50–5.59]	54	-3.033	0.002
D2	1.97[0.98–5.35]	26	0.70[0.34–1.60]	156	-14.125	< 0.001
IL-6	98.04[25.89-353.43]	172	14.08[4.83–44.83]	1377	-11.827	< 0.001
**Treatment strategy**						
Untreated	166(46.6)	NA	1231(59.0)	NA	20.941	< 0.001
Low-molecular-weight heparin	188(52.8)		835(40.0)			
Warfarin	1(0.3)		15(0.7)			
Novel oral anticoagulant	1(0.3)		7(0.3)			
glucocorticoid	204(57.3)	NA	771(36.9)	NA	52.672	< 0.001
**Anti-COVID-19 drug**						
Untreated	146(41.0)	NA	1223(58.6)	NA	45.208	< 0.001
Azvudine	158(44.4)	NA	674(32.3)	NA		
Nirmatrelvir/Ritonavir	32(9.0)	NA	146(7.0)	NA		
Azvudine + Nirmatrelvir/Ritonavir	20(5.6)	NA	45(2.2)	NA		
Bleeding event	37(10.4)	NA	28(1.3)	NA	96.269	< 0.001

COVID: coronavirus disease 2019; BMI: body mass index; CVD: cardiovascular disease; DM: diabetes mellitus; CLD: chronic lung disease; CRD: chronic renal disease; AID: autoimmune disease; ICU: intensive care unit; WBC: white blood cell; Neu: neutrophil; Lym: lymphocytes; NLR: neutrophil/lymphocyte ratio; Plt: platelets; sCRP: hypersensitive C-reactive protein; Hs-cTnT: high-sensitivity cardiac troponin T; Cr: creatinine; Alb: albumin; LDH: lactate dehydrogenase; CK: creatine kinase; BNP: brain natriuretic peptide; Fib: fibrinogen; D2: D-dimer; IL-6: interleukin-6; VTE: venous thromboembolism; NA: not applicable

### Comparison of clinical data in COVID-19 pneumonia patients with VTE between mortality and survival groups and exploration of risk factors for all-cause mortality

The results indicated that among COVID-19 pneumonia patients with VTE, levels of sCRP, Hs-cTnT, LDH, CK and IL-6 were significantly higher in mortality group than in survival group (*P* < 0.05) (Table 3). The proportion of patients with ICU admission or without anticoagulant therapy was significantly greater in mortality group than in survival group (*P* < 0.05) (Table 3).

**Fig. 5:**
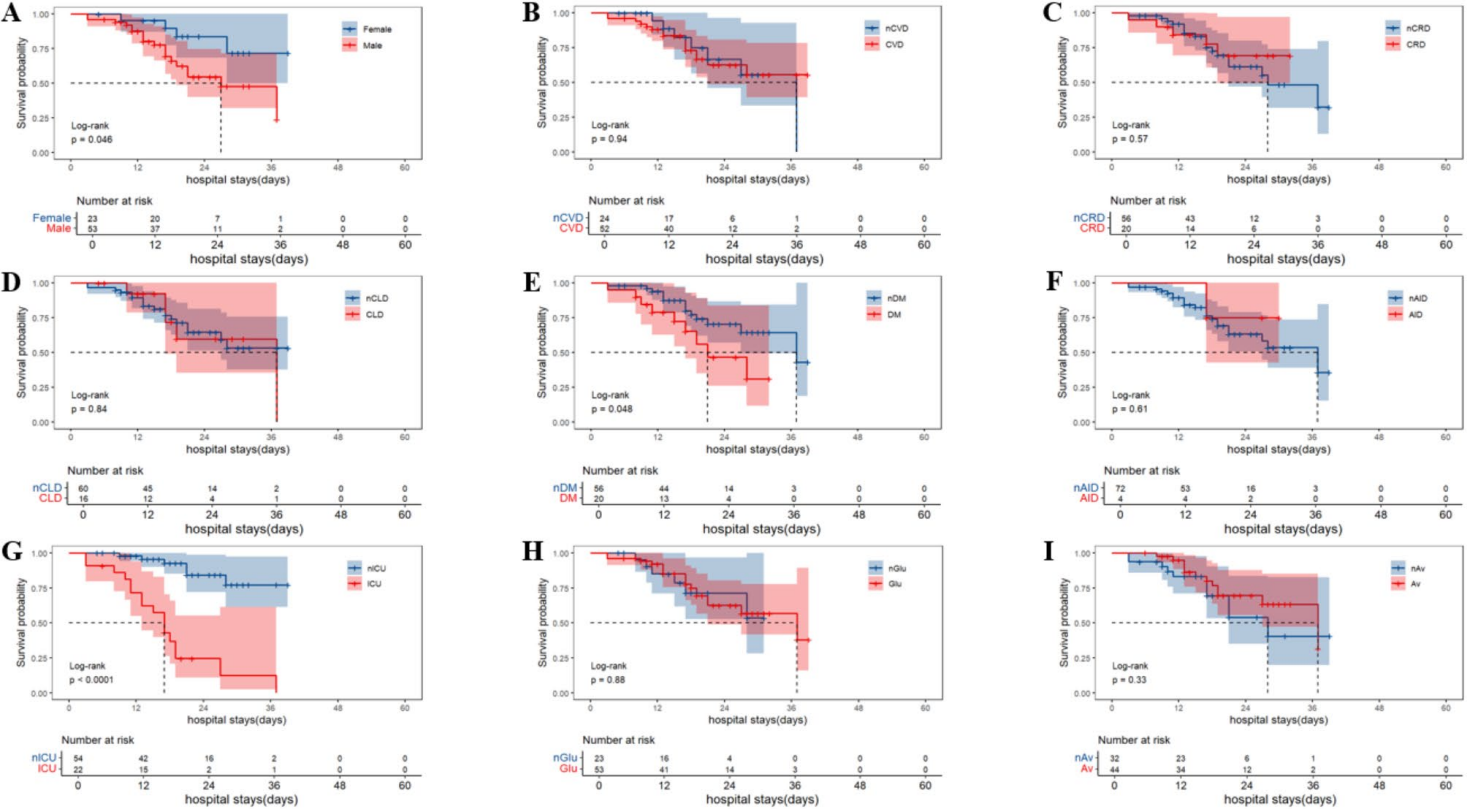
The effect of various factors on mortality in COVID-19 pneumonia patients with VTE by Kaplan-Meier curves and Log-rank test (Unadjusted) (**A**: Gender; **B**: CVD; **C**: CRD; **D**: CLD; **E**: DM; **F**: AID; **G**: ICU; **H**: Glu; **I**: Av) CVD: cardiovascular disease; CLD: chronic lung disease; CRD: chronic renal disease; DM: diabetes mellitus; AID: autoimmune disease; ICU: intensive care unit; Glu: glucocorticoid; Av: antiviral; VTE: venous thromboembolism; COVID: coronavirus disease 2019

Survival curve analysis demonstrated significant differences in mortality rates among COVID-19 pneumonia patients with VTE regarding male, DM and ICU admission (Log-rank test, *P* < 0.05) (Fig. 5A-I). By Kaplan-Meier curve (Fig. 5A-I) and Schoenfeld residual method (Supplementary Fig. 2), all target variables satisfied the proportional hazards assumption.

**Fig. 6:**
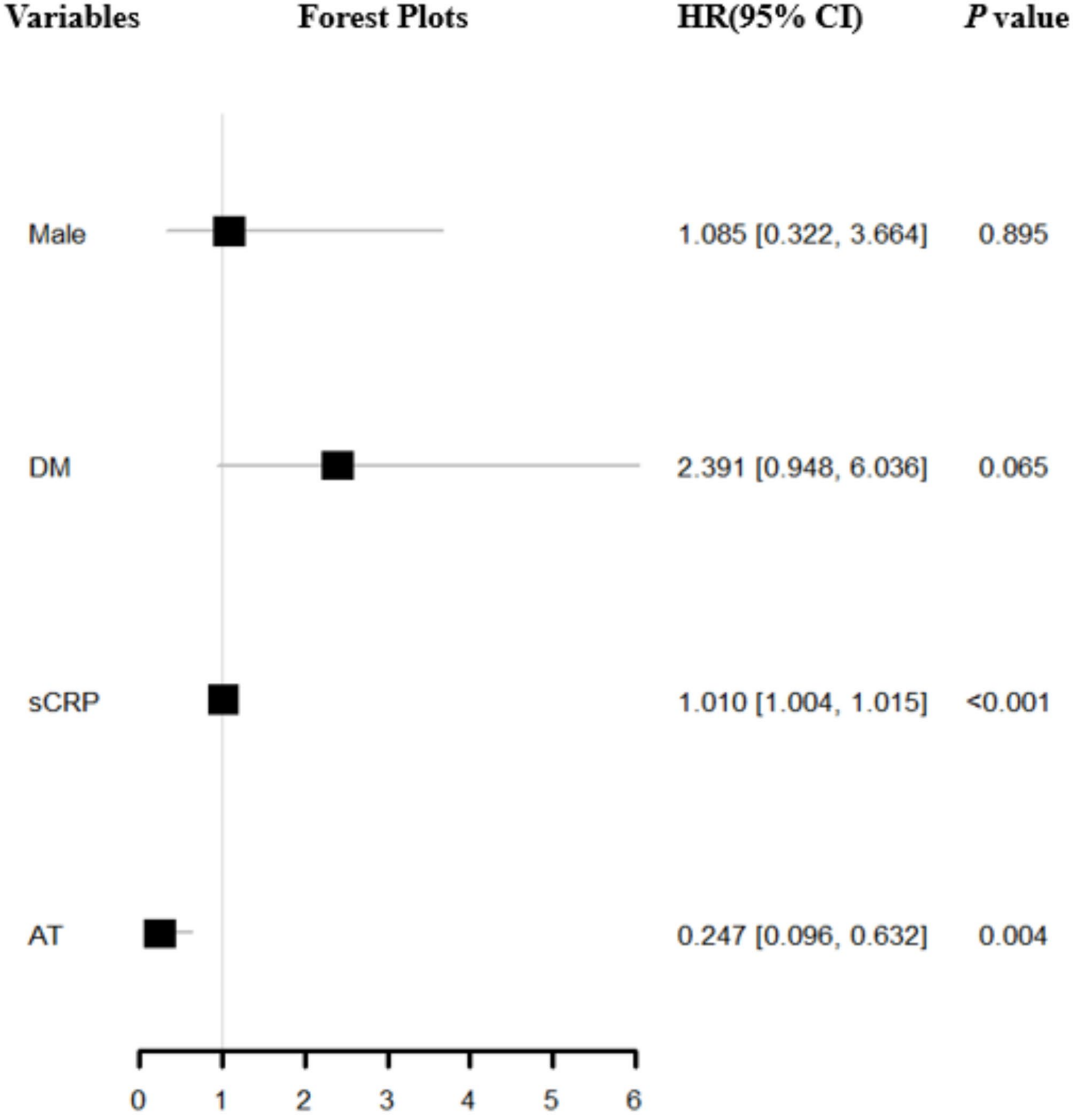
Forest Plots of Multivariate Cox Proportional Hazards Regression for in-hospital All-Cause Mortality in COVID-19 pneumonia Patients with VTE DM: diabetes mellitus; sCRP: hypersensitive C-reactive protein; AT: anticoagulant therapy; COVID: coronavirus disease 2019; VTE: venous thromboembolism

Eligible potential variables were incorporated into a multivariate Cox regression analysis, which revealed that sCRP (HR = 1.010, 95%CI= [1.004,1.015], *P* < 0.001), AT (HR = 0.247, 95%CI= [0.096,0.632], *P* = 0.004) were risk factors for in-hospital all-cause mortality in COVID-19 pneumonia patients with VTE (Fig. 6).

**Table 3:** Clinical characteristics and laboratory results of poor prognosis in COVID-19 pneumonia patients with VTE

	Mortality group (*n* = 23)	Missing data	Survival group (*n* = 53)	Missing data	t/Z/χ^2^	*P*
**General data**						
Age	81[72–86]	NA	80[70.5–85.5]	NA	-0.357	0.721
Male	19(82.6)	NA	34(64.2)	NA	2.589	0.108
BMI	24.30 ± 2.89	7	23.48 ± 3.76	10	-0.788	0.434
Smoking history	6(26.1)	NA	17(32.1)	NA	0.273	0.602
Prior VTE history	3(13)	NA	1(1.9)	NA	2.097	0.149
Surgical history within 3 months	1(4.3)	NA	0(0)	NA	$	0.303
CVD	16(69.6)	NA	36(67.9)	NA	0.020	0.888
DM	9(39.1)	NA	11(20.8)	NA	2.793	0.095
CLD	5(21.7)	NA	11(20.8)	NA	0.009	0.923
CRD	5(21.7)	NA	15(28.3)	NA	0.356	0.551
AID	1(4.3)	NA	3(5.7)	NA	0.055	0.814
ICU admission	17(73.9)	NA	5(9.4)	NA	32.422	< 0.001
Hospital stays (Day)	16.0 ± 7.8	NA	19.2 ± 8.4	NA	1.567	0.121
**Laboratory data**						
WBC	7.34[5.26–9.69]	NA	6.41[5.29–8.91]	NA	-0.475	0.635
Neu	6.08[4.48–8.93]	NA	5.02[3.74–8.20]	NA	-0.944	0.345
Lym	0.69[0.33–0.81]	NA	0.66[0.49–0.94]	NA	-1.080	0.280
NLR	5.64[3.23–9.79]	NA	4.31[2.85–7.31]	NA	-1.362	0.173
Plt	131[98–172]	NA	161[116–212]	NA	-1.849	0.064
sCRP	116.4[41.2-169.8]	NA	41.5[21.7–78.8]	NA	-3.126	0.002
Hs-cTnT	70.4[18.1–225.0]	NA	21.5[17.6–32.5]	2	-2.202	0.028
Urea	9.67[6.77–19.26]	NA	7.33[5.05–11.10]	NA	-1.871	0.061
Cr	91.0[72.2–154.0]	NA	80.0[63.5–128.0]	NA	-0.842	0.400
Alb	32.5 ± 5.4	NA	33.0 ± 5.5	NA	0.360	0.720
LDH	406[353–525]	NA	290[231–396]	NA	-3.681	< 0.001
CK	122[86–180]	2	70[44–147]	10	-2.202	0.028
BNP	262.9[76.0-713.4]	NA	88.8[39.9-230.8]	NA	-1.939	0.052
Fib	4.80 ± 1.92	NA	4.31 ± 1.16	NA	-1.347	0.182
D2	1.99[0.88–6.24]	NA	1.90[0.96–5.13]	NA	-0.073	0.941
IL-6	117.24[36.98-281.41]	7	15.27[6.65–59.56]	22	-3.525	< 0.001
**Treatment strategy**						
Untreated	8(34.8)	NA	7(13.2)	NA	4.713	0.030
Low-molecular-weight heparin	15(65.2)		46(86.8)			
Warfarin	0(0)		0(0)			
Novel oral anticoagulant	0(0)		0(0)			
glucocorticoid	17(73.9)	NA	36(67.9)	NA	0.273	0.602
**Anti-COVID-19 drug**						
Untreated	11(47.8)	NA	21(39.6)	NA	$	0.271
Azvudine	7(30.4)	NA	26(49.1)	NA		
Nirmatrelvir/Ritonavir	3(13.0)	NA	5(9.4)	NA		
Azvudine + Nirmatrelvir/Ritonavir	2(8.7)	NA	1(1.9)	NA		
Bleeding event	1(4.3)	NA	4(7.5)	NA	< 0.001	0.989

$: Fisher’s exact test; COVID: coronavirus disease 2019; BMI: body mass index; CVD: cardiovascular disease; DM: diabetes mellitus; CLD: chronic lung disease; CRD: chronic renal disease; AID: autoimmune disease; ICU: intensive care unit; WBC: white blood cell; Neu: neutrophil; Lym: lymphocytes; NLR: neutrophil/lymphocyte ratio; Plt: platelets; sCRP: hypersensitive C-reactive protein; Hs-cTnT: high-sensitivity cardiac troponin T; Cr: creatinine; Alb: albumin; LDH: lactate dehydrogenase; CK: creatine kinase; BNP: brain natriuretic peptide; Fib: fibrinogen; D2: D-dimer; IL-6: interleukin-6; VTE: venous thromboembolism; NA: not applicable

### Comparison of the effect of anticoagulant therapy on mortality between COVID-19 pneumonia patients with VTE and those with nonvte

**Fig. 7:**
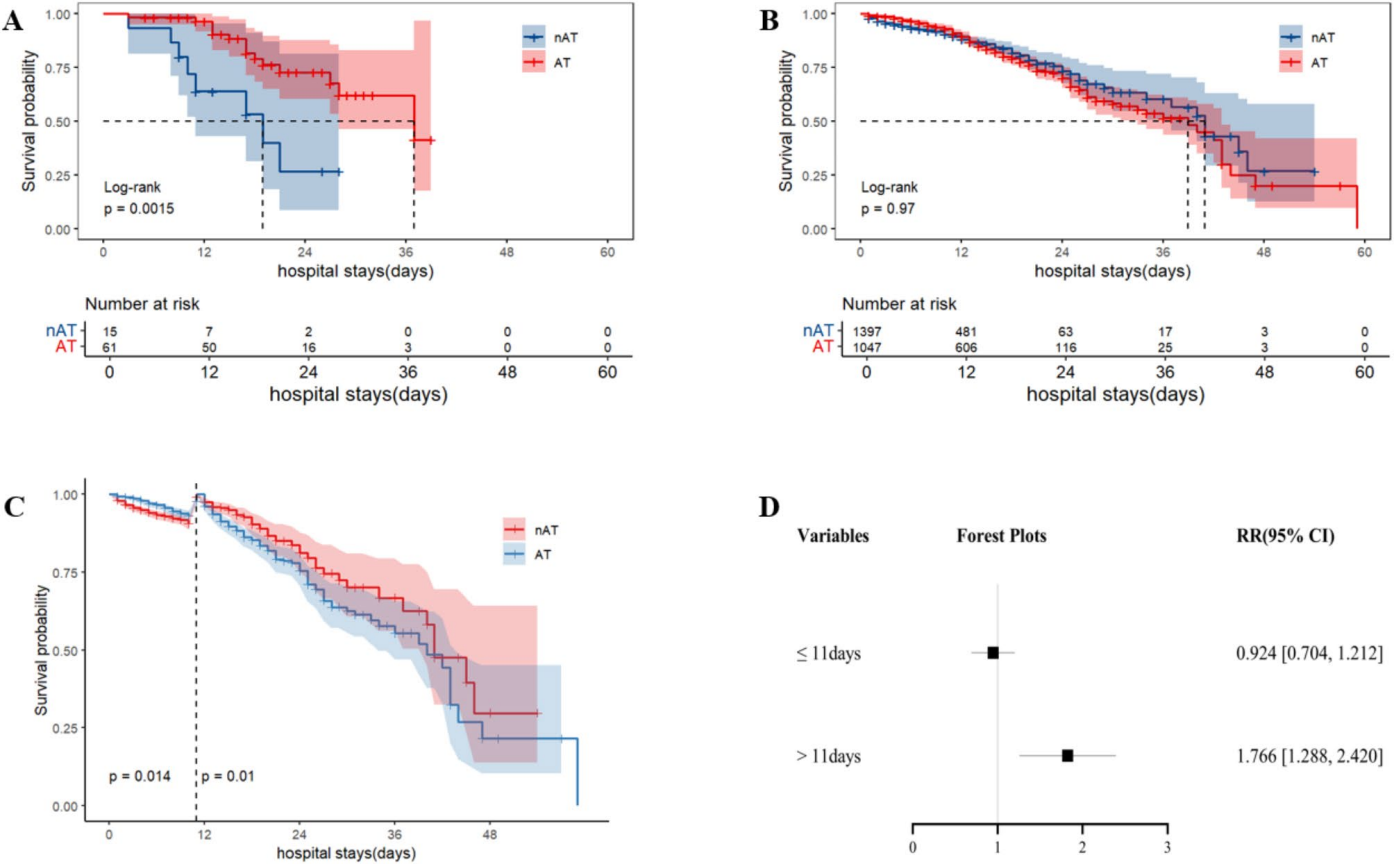
Effect of anticoagulant therapy on mortality in patients with COVID-19 pneumonia **A**: Effect of anticoagulant therapy on mortality in COVID-19 pneumonia patients with VTE; **B**: Effect of anticoagulant therapy on mortality in COVID-19 pneumonia patients with nonVTE; **C**: Effect of anticoagulant therapy on mortality in COVID-19 pneumonia patients with nonVTE before and after 11 days of hospitalization by Landmark survival analysis; **D**: Effect of hospital stays on mortality in COVID-19 pneumonia patients with nonVTE receiving anticoagulant therapy AT: anticoagulant therapy; VTE: venous thromboembolism; COVID: coronavirus disease 2019

Survival curve analysis indicated that anticoagulant therapy had a statistically significant impact on mortality in COVID-19 pneumonia patients with VTE (Log-rank test, *P* = 0.0015) (Fig. 7A). In contrast, there was no statistically significant effect of anticoagulant therapy on mortality in COVID-19 pneumonia patients with nonVTE (Log-rank test, *P* = 0.97) (Fig. 7B). Landmark analysis results showed that for the hospital stays of 11 days or less, the difference in the impact of anticoagulant therapy on mortality was statistically significant in COVID-19 pneumonia patients with nonVTE (≤ 11days, *P* = 0.014; > 11days, *P* = 0.01) (Fig. 7C).

Among patients with nonVTE, the risk of death was lower in patients receiving anticoagulant therapy than those without anticoagulant therapy (RR = 0.924, 95%CI= [0.704,1.212]) when the length of hospital stays was ≤ 11days; however, when the length of hospital stays was > 11 days, those receiving anticoagulant therapy had a higher risk of death compared to those not receiving anticoagulant therapy (RR = 1.766, 95% CI= [1.288,2.420]) (Fig. 7D).

### Comparison between anticoagulant therapy and non-anticoagulant therapy in COVID-19 pneumonia patients with nonvte

The levels of age, hospital stays, peripheral blood WBC, Neu, NLR, sCRP, Hs-cTnT, Urea, Cr, LDH, BNP, Fib, D2 and IL-6 in COVID-19 pneumonia patients with nonVTE were significantly higher in anticoagulant group than non-anticoagulant group (*P* < 0.05) (Table 4). The levels of Lym and Alb decreased significantly in anticoagulant group compared to non-anticoagulant group (*P* < 0.05) (Table 4). Additionally, the proportions of male patients, CVD, CLD, ICU admission, use of Glu, use of anti-COVID-19 drugs and death were significantly higher in anticoagulant group compared to those in non-anticoagulant group (*P* < 0.05) (Table 4).

**Table 4:** Comparison of clinical characteristics and laboratory results in COVID-19 pneumonia patients with nonvte between anticoagulant therapy group and non-anticoagulant therapy group

	Anticoagulant group				Non-anticoagulant group				*P* ^&^
	Total (*n* = 1047)	≤ 11 days(*n* = 441)^a^	> 11 days(*n* = 606)^b^	*P* ^#^	Total (*n* = 1397)	≤ 11 days(*n* = 916)	> 11 days(*n* = 481)	*P* ^^^	
**General data**									
Age	77[66–84]	77[66–83]^***^	78[67–85]^***^	0.030	69[56,80]	68[54–79]	71[57–82]	0.002	< 0.001
Male	692(66.1)	285(64.6)^**^	407(67.2)	0.329	825(59.1)	520(56.8)	305(63.4)	0.016	< 0.001
BMI	23.36[21.08–25.95]	23.46[21.20–26.00]	23.24[20.96–25.95]^*^	0.642	23.41[20.81–25.81]	23.47[21.09–26.01]	23.08[20.30-25.39]	0.005	0.472
Smoking history	250(23.9)	109(24.7)	141(23.3)	0.587	311(22.3)	188(20.5)	123(25.6)	0.031	0.347
Prior VTE history	14(1.3)	4(0.9)	10(1.7)	0.301	13(0.9)	5(0.5)	8(1.7)	0.076	0.341
Surgical history within 3 months	6(0.6)	3(0.7)	3(0.5)^*^	1.000	18(1.3)	8(0.9)	10(2.1)	0.058	0.076
CVD	684(65.3)	263(59.6)^**^	421(69.5)^***^	< 0.001	749(53.6)	473(51.6)	276(57.4)	0.041	< 0.001
DM	290(27.7)	109(24.7)	181(29.9)	0.066	391(28.0)	247(27.0)	144(29.9)	0.240	0.874
CLD	252(24.1)	82(18.6)	170(28.1)^*^	< 0.001	264(18.9)	157(17.1)	107(22.2)	0.021	0.002
CRD	232(22.2)	76(17.2)	156(25.7)	0.001	279(20.0)	171(18.7)	108(22.5)	0.093	0.188
AID	66 (6.3)	17(3.9)	49(8.1)	0.005	65(4.7)	37(4.0)	28(5.8)	0.133	0.073
ICU admission	204(19.5)	91(20.6)	113(18.6)	0.423	147(10.5)	104(11.4)	43(8.9)	0.162	< 0.001
Hospital stays (Day)	13[9–18]	8[6–10]^***^	17[14–21]^***^	< 0.001	9.0[6.0–13.0]	7(5–9)	15[13–19]	< 0.001	< 0.001
Anticoagulant duration (Day)	8[5–13]	6[3–8]	12[8–16]	< 0.001	NA	NA	NA	NA	NA
**Laboratory data**									
WBC	6.49[4.84–8.91]	6.64[5.02–8.96]^***^	6.43[4.79–8.91]	0.292	6.16[4.56–8.23]	6.01[4.53–7.96]	6.48[4.60–8.65]	0.054	< 0.001
Neu	5.05[3.48–7.44]	5.00[3.48–7.57]^***^	5.13[3.48–7.42]^*^	0.762	4.37[2.96–6.48]	4.18[2.91–6.22]	4.57[3.11–7.04]	0.005	< 0.001
Lym	0.77[0.50–1.11]	0.85[0.56–1.25]^***^	0.72[0.47–1.02]^***^	< 0.001	0.95[0.60–1.40]	0.99[0.62–1.44]	0.89[0.51–1.30]	0.003	< 0.001
NLR	6.60[3.67–2.35]	5.62[3.36–11.23]^***^	7.06[4.03–13.27]^***^	0.001	4.51[2.63–9.22]	4.19[2.41–8.29]	5.27[2.93–10.87]	< 0.001	< 0.001
Plt	174[123–243]	182[129–260]	171[122–232]	0.012	176[127–237]	178[128–239]	169[125–227]	0.185	0.734
sCRP	44.5[13.0-88.1]	35.8[9.9–78.3]^***^	51.3[16.8–98.2]^***^	< 0.001	18.1[4.8–56.6]	15.2[3.9–51.3]	27.5[7.8–70.6]	< 0.001	< 0.001
Hs-cTnT	20.1[11.0-39.9]	16.3[8.4–30.3]^*^	22.9[13.8–42.8]^***^	< 0.001	14.8[7.3–33.9]	13.0[6.7–32.0]	18.4[8.7–36.8]	< 0.001	< 0.001
Urea	6.96[5.10–10.60]	6.70[5.06–9.89]^***^	7.26[5.10–11.0]^*^	0.161	6.10[4.40–9.29]	5.83[4.21–8.80]	6.65[4.82–9.83]	0.001	< 0.001
Cr	87.2[66.9-120.7]	86.0[65.8–112.0]^**^	89.0[67.6-129.1]^*^	0.053	80.0[62.2-109.7]	78.0[61.6-107.1]	84.0[63.9-115.9]	0.031	< 0.001
Alb	34.7[31.3–38.0]	35.5[32.0-38.5]^***^	34.4[30.6–37.5]^***^	< 0.001	37.2[33.2–40.4]	37.9[33.8–40.8]	36.0[32.4–39.3]	< 0.001	< 0.001
LDH	293[232–381]	287[221–366]^***^	296[237–390]^***^	0.018	239[197–308]	233[196–301]	253[200–323]	0.004	< 0.001
CK	73[43–158]	75[43–161]	73[43–157]	0.870	75[47–138]	73[47–132]	75[46–142]	0.980	0.947
BNP	69.7[27.8-150.7]	61.1[21.6-156.3]^**^	74.9[31.2-148.1]^**^	0.045	47.1[16.6-139.4]	43.4[15.8–131.0]	58.2[19.3–153.0]	0.081	< 0.001
Fib	4.86[3.97-6.00]	4.85[3.86–5.83]^***^	4.87[4.02–6.18]^***^	0.072	4.30[3.28–5.42]	4.23[3.24–5.35]	4.46[3.46–5.68]	0.011	< 0.001
D2	1.01[0.53–2.41]	0.92[0.45–2.26]^***^	1.08[0.57–2.45]^***^	0.085	0.65[0.31–1.60]	0.62[0.28–1.56]	0.73[0.38–1.68]	0.017	< 0.001
IL-6	26.65[6.95–83.06]	18.62[5.28–69.42]^*^	31.40[8.73–93.33]^*^	0.005	14.43[4.93–47.23]	11.32[3.83–43.75]	19.31[8.30-49.44]	0.007	< 0.001
glucocorticoid	614(58.6)	224(50.8)	390(64.4)	< 0.001	361(25.8)	188(20.5)	173(36.0)	< 0.001	< 0.001
**Anti-COVID-19 drug**									
Untreated	452(43.2)	201(45.6)	251(41.4)	0.030	917(65.6)	626(68.3)	291(60.5)	0.260	< 0.001
Azvudine	434(41.5)	178(40.4)	256(42.2)		398(28.5)	240(26.2)	158(32.8)		
Nirmatrelvir/Ritonavir	113(10.8)	51(11.6)	62(10.2)		65(4.7)	41(4.5)	24(5.0)		
Azvudine + Nirmatrelvir/Ritonavir	48(4.6)	11(2.5)	37(6.1)		17(1.2)	9(1.0)	8(1.7)		
Bleeding event	34(3.2)	5(1.1)	29(4.8)	0.001	31(2.2)	16(1.7)	15(3.1)	0.098	0.118
In-hospital mortality	190(18.1)	81(18.4)^**^	109(18.0)^***^	0.875	166(11.9)	117(12.8)	49(10.2)	0.156	< 0.001

COVID: coronavirus disease 2019; BMI: body mass index; CVD: cardiovascular disease; DM: diabetes mellitus; CLD: chronic lung disease; CRD: chronic renal disease; AID: autoimmune disease; ICU: intensive care unit; WBC: white blood cell; Neu: neutrophil; Lym: lymphocytes; NLR: neutrophil/lymphocyte ratio; Plt: platelets; sCRP: hypersensitive C-reactive protein; Hs-cTnT: high-sensitivity cardiac troponin T; Cr: creatinine; Alb: albumin; LDH: lactate dehydrogenase; CK: creatine kinase; BNP: brain natriuretic peptide; Fib: fibrinogen; D2: D-dimer; IL-6: interleukin-6; VTE: venous thromboembolism; NA: not applicable

#: in anticoagulant group, ≤ 11 days vs. > 11 days; ^: in non-anticoagulant group, ≤ 11 days vs. > 11 days; &: anticoagulant group vs. non-anticoagulant group; ^a^: when the hospital stays were ≤ 11 days, anticoagulant group vs. non-anticoagulant group; ^b^: when the hospital stays were > 11 days, anticoagulant group vs. > non-anticoagulant group; *: *P* < 0.05; **: *P* < 0.01; ***: *P* < 0.0001

### Factors associated with hospital stays > 11 days in COVID-19 pneumonia patients with nonvte receiving anticoagulant therapy and the effect of different anticoagulants on patient mortality

**Fig. 8:**
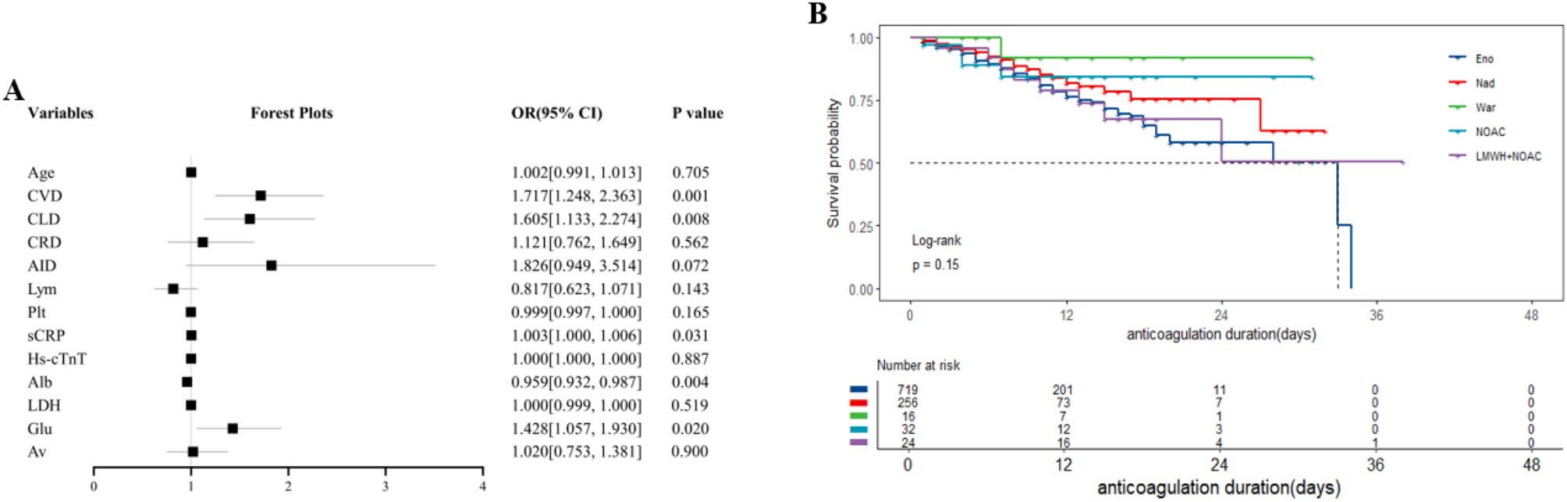
**A** Forest plot of factors associated with hospital stays > 11 days in COVID-19 pneumonia patients with nonVTE receiving anticoagulant therapy. **B** Effect of different anticoagulants on mortality of COVID-19 pneumonia patients with nonVTE CVD: cardiovascular disease; CLD: chronic lung disease; CRD: chronic renal disease; AID: autoimmune disease; Lym: lymphocytes; Plt: platelets; sCRP: hypersensitive C-reactive protein; Hs-cTnT: high-sensitivity cardiac troponin T; Alb: albumin; LDH: lactate dehydrogenase; Glu: glucocorticoid; Av: antiviral; VTE: venous thromboembolism; COVID: coronavirus disease 2019; Eno: enoxaparin; Nad: nadroparin; War: warfarin; NOAC: novel oral anticoagulants; LMWH: low-molecular-weight heparin

Multivariate binary logistic regression analysis was performed on potential variables with statistical differences using input method. The results demonstrated that CVD (OR = 1.717, 95%CI= [1.248,2.363], *P* = 0.001), CRD (OR = 1.605, 95%CI= [1.133,2.274], *P* = 0.008), sCRP (OR = 1.003, 95%CI= [1.000,1.006], *P* = 0.031), Alb (OR = 0.959, 95%CI = [0.932,0.987], *P* = 0.004) and glucocorticoid (OR = 1.428, 95%CI= [1.057,1.930], *P* = 0.020) were independent factors associated with hospital stays > 11 days in anticoagulant group (Fig. 8A).

Survival curve analysis suggested that there was no statistically significant difference in mortality among COVID-19 pneumonia patients with nonVTE using different anticoagulants (Log-rank test, *P* = 0.15) (Fig. 8B).

## Discussion

With the decreasing virulence of SARS-CoV-2 variants and the acquired immunity brought about by vaccination, the COVID-19 pandemic is gradually coming under control globally. The in-hospital mortality among COVID-19 pneumonia patients has declined compared to the previous period, but the risk of death within 60 days after hospitalization remains high [23]. Identifying risk factors associated with in-hospital mortality can facilitate early identification of high-risk patients. Nonetheless, some potential risk factors may vary with hospital stays. Anticoagulant therapy can effectively reduce in-hospital mortality in patients, however, there is still a lack of detailed research data about the impact of anticoagulation therapy on mortality of COVID-19 pneumonia patients during the entire hospitalization period. Therefore, our study systematically summarized the risk factors of mortality in COVID-19 pneumonia patients, and further found that those receiving prophylactic anticoagulants in the early stage of hospitalization exhibited significantly lower mortality rates compared to those with non-anticoagulant therapy. However, the effectiveness of anticoagulant therapy in reducing mortality cannot be sustained as hospital stays extends, which was influenced by factors such as inflammatory states, comorbidities, organ damage and adverse events during the treatment process.

Our study reported that COVID-19 pneumonia patients with VTE were significantly older and had longer hospital stays compared to patients with nonVTE. These patients also had a higher proportion of prior VTE history, ICU admission and abnormal laboratory results indicating inflammatory responses and organ dysfunction. These findings are only partially consistent with previous studies due to our exclusion of malignancy, confirmed VTE cases and patients without pneumonia from the analysis [24–26]. No significant difference was observed in in-hospital mortality between COVID-19 pneumonia patients with VTE and nonVTE after anticoagulant therapy. It may be attributed to the early implementation of prophylactic anticoagulation, which effectively reduced the risk of in-hospital mortality in both groups [26, 27]. However, the in-hospital mortality of COVID-19 pneumonia patients with VTE receiving non-anticoagulant therapy was significantly higher than that of patients with nonVTE, underscoring the critical importance of anticoagulant treatment in improving the prognosis of patients with nonVTE.

When exploring the potential factors influencing all-cause mortality during hospitalization in COVID-19 pneumonia patients, we identified male, low levels of BMI, Lym, Plt and Alb, and elevated LDH level at admission as predictors of in-hospital mortality among COVID-19 pneumonia patients with nonVTE. It is noteworthy that these laboratory indicators reflect the severity of COVID-19 pneumonia and are of considerable significance in forecasting patient prognosis during hospitalization [28]. Previous studies proved that male and low levels of BMI, Plt, Lym as well as Alb were associated with higher mortality risks [29–31]. However, these studies involved all COVID-19 patients; since COVID-19 pneumonia represents a more severe disease state, our findings confirm that these markers also serve as risk factors affecting the prognosis of COVID-19 pneumonia patients. This study also focused on the prognosis of COVID-19 pneumonia patients with VTE and revealed that elevated sCRP levels at admission can act as indicators of in-hospital all-cause mortality in COVID-19 pneumonia patients with VTE. As a biomarker, sCRP can reflect the body’s inflammatory status, and inflammation is a crucial factor for the occurrence of VTE in COVID-19. Elevated sCRP levels also signify the risk of ICU admission, severe disease, organ dysfunction and mortality in COVID-19 patients [32]. Nevertheless, it remains unverified whether sCRP has predictive significance for mortality risk throughout the hospitalization period in COVID-19 pneumonia patients with nonVTE. This is because the effect of sCRP on mortality varies with the length of hospital stays, and does not conform to the proportional hazards assumption, thus not being included in the regression analysis. Additionally, although anticoagulant therapy can significantly improve prognosis during hospitalization for COVID-19 pneumonia patients with VTE, there is a potential risk of bleeding [33], which is the direct cause hindering the use of anticoagulants and increasing patient mortality.

Further research indicated that anticoagulant therapy significantly reduced in-hospital all-cause mortality in patients hospitalized for 11 days or less. This aligns with prior studies suggesting that prophylactic anticoagulation effectively decreases mortality in COVID-19 patients [34]. However, the landmark test reflected that when the length of hospital stays exceeded 11 days, prophylactic anticoagulation did not reduce mortality risk. Based on the Padua score, patients at high risk for thrombosis received prophylactic anticoagulation. Patients in anticoagulant group were characterized by older age, longer hospital stays, increased levels of indicators related to inflammation and organ damage, and high proportion of cardiac and pulmonary comorbidities, all of which were potential factors contributing to increased mortality risk. Iam-Arunthai K et al. noted that higher thrombotic risk scores correlated with increased mortality in COVID-19 patients [35]. Therefore, while anticoagulant therapy improves short-term prognosis in COVID-19 patients, its benefits diminish with extended hospital stays due to inflammatory states. Attention should be paid to patients in anticoagulant group with prolonged hospital stays (over 11 days). Our study also explored factors associated with longer hospital stays in anticoagulant group; the results demonstrated that COVID-19 pneumonia combined with CVD and CLD was related to prolonged hospitalization, which was consistent with the conclusions of previous studies [36, 37]. In addition, elevated sCRP and decreased Alb levels were linked with increased hospital stays, highlighting the need for interventions addressing inflammation and nutritional status such as low protein. Antiviral therapy did not affect hospital stays, but the use of glucocorticoid extended the length of hospital stays. Though glucocorticoid is generally considered effective in controlling inflammation and improving hospitalization outcomes, a meta-analysis on COVID-19 found that only very high doses of methylprednisolone (> 80 mg/day, but ≤ 200 mg/day) were relative to shorter hospital stays— a condition rarely met in this study [38]. Administering glucocorticoid after hospitalization means active inflammation and severe disease; concomitant use may increase risks of gastrointestinal bleeding, elevated blood sugar and pressure, and other adverse effects, leading to prolonged hospital stays [39]. Given the potential relationship between extended hospital stays and increased mortality risk, future research should focus on COVID-19 pneumonia patients hospitalized beyond 11 days. Finally, the anticoagulant type or duration had no effect on in-hospital mortality in COVID-19 pneumonia patients. Previous studies showed that anticoagulant type, dose or duration did not effectively improve the poor prognosis in COVID-19 pneumonia patients [40–42].

Our study has several limitations. First, this is a single-center retrospective design, and there is an inherent potential risk of making type 1 error in this study, highlighting the necessity for future verification of all results through multi-center, large-sample prospective cohort studies or randomized controlled trials; furthermore, the study design should ensure an appropriate balance between groups and the comparability of data. If necessary, more robust statistical methods should be employed to minimize the likelihood of errors. Second, we were unable to perform CTPA and intravenous color ultrasonography on all hospitalized patients with COVID-19 pneumonia; consequently, some patients with VTE may not be identified, potentially influencing sample size and introducing selection bias. Third, due to the varying impact of disease severity on inflammation, thrombosis and mortality risk, future research should place greater emphasis on subgroup analysis based on the severity of COVID-19 pneumonia. Fourth, due to the retrospective study design, we were unable to obtain detailed information regarding whether patients had received vaccinations, the types of vaccines administered, and the number of vaccinations after reviewing their electronic medical records. Consequently, we could not analyze the relationship between vaccination and the use of remdesivir in conjunction with anticoagulant therapy and poor prognosis in hospitalized patients with COVID-19 pneumonia. Finally, in addition to the type, dose and duration of anticoagulants, it is also essential to consider factors such as patient age, inflammatory status, comorbidities, organ dysfunction, types of anti-COVID-19 drugs and hospital stays when exploring the effectiveness and prognostic impact of anticoagulant therapy in COVID-19 pneumonia patients.

## Conclusion

In conclusion, our study found no significant difference in mortality between hospitalized COVID-19 pneumonia patients with VTE and nonVTE when receiving prophylactic anticoagulation. Among COVID-19 pneumonia patients with nonVTE, in-hospital all-cause mortality correlated with factors such as male, BMI, peripheral blood lymphocytes, platelets, albumin and LDH at admission. Conversely, among COVID-19 pneumonia patients with VTE, in-hospital all-cause mortality was associated with peripheral blood sCRP levels at admission and anticoagulant therapy. Prolonged hospital stays (> 11 days) may limit the effectiveness of prophylactic anticoagulation on lower in-hospital mortality for COVID-19 pneumonia patients with nonVTE. Prolonged hospital stays were related to cardiovascular disease, chronic renal disease, peripheral blood sCRP, albumin and use of glucocorticoid at admission. These findings enhance clinical understanding of the prognostic factors influencing hospitalized COVID-19 pneumonia patients, emphasizing the importance of early management and intervention for relevant factors, and provide more evidence for future research on anticoagulant strategy in COVID-19 pneumonia.

## Electronic supplementary material

Below is the link to the electronic supplementary material.

Supplementary Material 1

## Data Availability

The original data presented in the study are included in the manuscript/Supplementary Material, further inquiries can be directed to the corresponding author.
