# Supplementary material for: Prognostic factors in hospitalized patients with COVID-19 pneumonia and effectiveness of prophylactic anticoagulant therapy: a single-center retrospective study
Source: BMC Infect Dis. 2025 Mar 3;25:303. doi: 10.1186/s12879-025-10666-3 (PMC11877778; doi:10.1186/s12879-025-10666-3)
Supplement: Supplementary file 1 — Supplementary Material 1 [file 12879_2025_10666_MOESM1_ESM.docx]

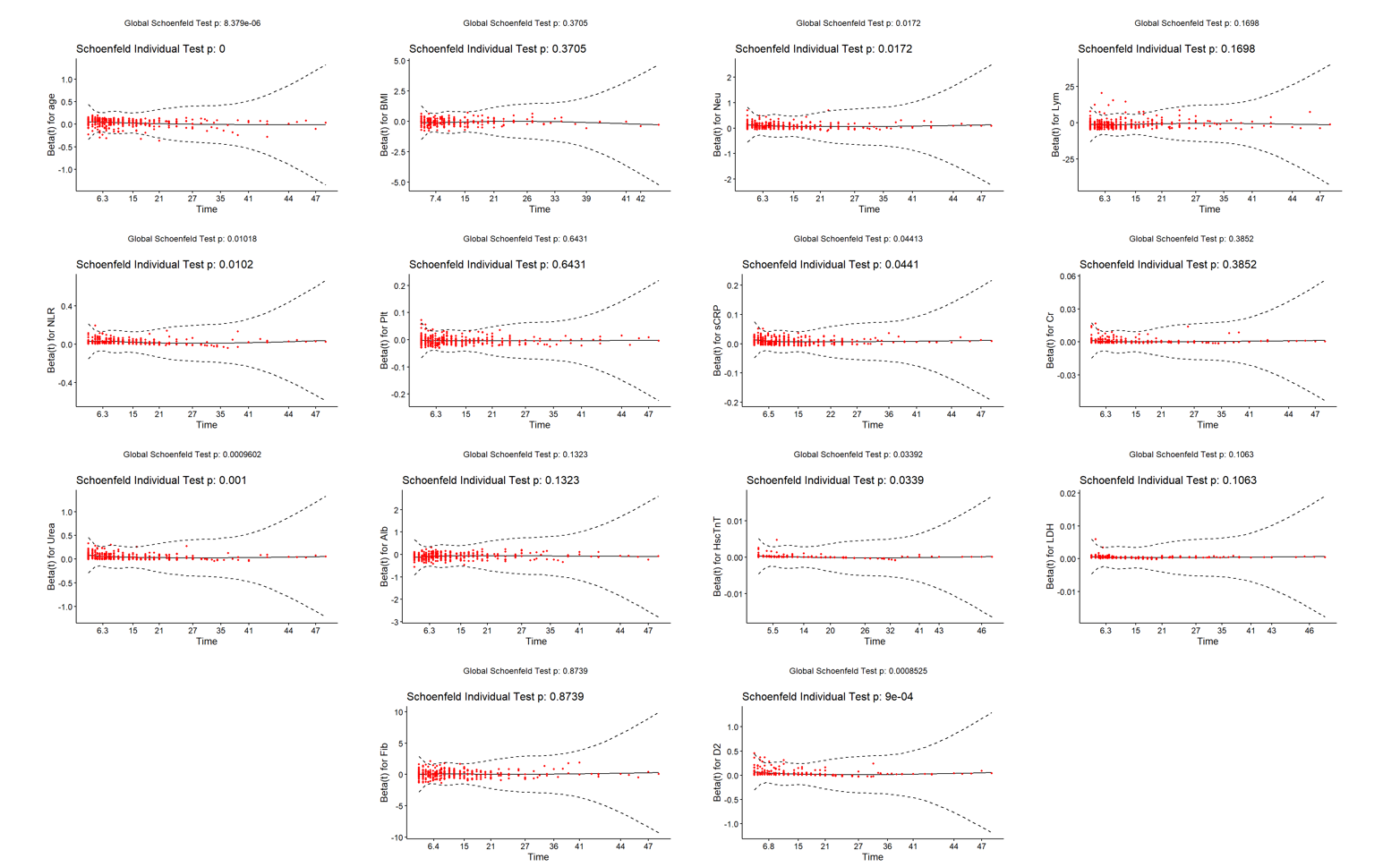


Supplementary Figure 1 Identification of continuous variables associated with in-hospital all-cause mortality in COVID-19 pneumonia patients with nonVTE using Schoenfeld residual method

BMI: body mass index; Neu: neutrophil; Lym: lymphocytes; NLR: neutrophil/lymphocyte ratio; Plt: platelets; Cr: creatinine; sCRP: hypersensitive C-reactive protein; Alb: albumin; Hs-cTnT: high-sensitivity cardiac troponin T; Fib: fibrinogen; D2: D-dimer; LDH: lactate dehydrogenase; COVID: coronavirus disease 2019; VTE: venous thromboembolism.


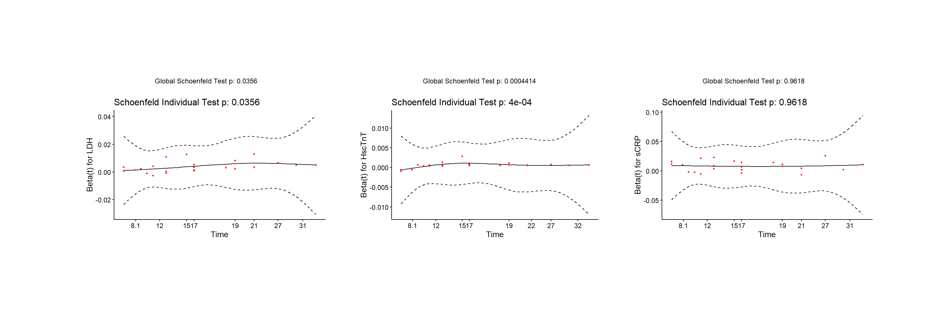


Supplementary Figure 2 Identification of continuous variables associated with in-hospital all-cause mortality in COVID-19 pneumonia patients with VTE using Schoenfeld residual method

sCRP: hypersensitive C-reactive protein; Hs-cTnT: high-sensitivity cardiac troponin T; LDH: lactate dehydrogenase; COVID: coronavirus disease 2019; VTE: venous thromboembolism.

Supplementary Table 1 Collinearity diagnosis of independent variables in multiple logistic regression analysis

| model | Collinearity Statistics | |
| --- | --- | --- |
|  | Tolerance | VIF |
| (constant) |  |  |
| BMI | 0.962 | 1.039 |
| Lym | 0.844 | 1.185 |
| Plt | 0.875 | 1.143 |
| Cr | 0.956 | 1.046 |
| Alb | 0.912 | 1.097 |
| LDH | 0.934 | 1.070 |
| Fib | 0.872 | 1.147 |

BMI: body mass index; Lym: lymphocytes; Plt: platelets; Cr: creatinine; Alb: albumin; Fib: fibrinogen; LDH: lactate dehydrogenase.
